# Case Report: Two adjacent ventral slots for treatment of multiple compressive cervical intervertebral disc protrusions in a British Shorthair cat

**DOI:** 10.3389/fvets.2026.1779092

**Published:** 2026-04-13

**Authors:** Lisa Castellano, Michał Mól

**Affiliations:** 1 Surgery Department, Davies Veterinary Specialists, Linnaeus Veterinary Limited, Mars Veterinary Health, Higham Gobion, United Kingdom; 2 Neurology and Neurosurgery Department, Paragon Veterinary Referrals, Wakefield, United Kingdom

**Keywords:** British Shorthair, double ventral slot, feline intervertebral disc disease, feline neurosurgery, non-ambulatory hemiparesis

## Abstract

An 8-year, 10-month-old female spayed British Shorthair cat was referred for progressive tetraparesis. At presentation, the cat was non-ambulatory with left-sided hemiparesis Cervical magnetic resonance imaging showed presence of extradural disc material causing moderate spinal cord compression at C3-C4 and C4-C5 levels, and mild compression at C5-C6 level. A double ventral slot surgery was performed at the C3-C4 and C4-C5 levels. Following an initial neurological deterioration, the cat made a steady recovery and was fully ambulatory 10 days postoperatively. At the 12-month follow-up, the cat showed only mild left pelvic limb monoparesis. Intervertebral disc disease is still rarely reported in cats, compared to other species. This report describes the use of two adjacent ventral slots to manage multilevel compressive cervical intervertebral disc disease in a British Shorthair cat, with its 12-month follow-up.

## Introduction

Intervertebral disc disease (IVDD) is a widely described disease in veterinary medicine, although feline IVDD is seldom reported (1–3). Data extrapolated from multiple post-mortem examinations indicate that IVDD is a frequent finding in asymptomatic feline patients (4, 5). However, the prevalence of symptomatic IVDD is very low, with data reporting it between 0.12 and 0.44% among the overall cat population (1, 6–8) and 4 to 5% in cats with spinal neurological signs (2, 3). Several types of IVDD have been described in cats: intervertebral disc extrusion (IVDE), intervertebral disc protrusion (IVDP), acute non-compressive nucleus pulposus extrusion (ANNPE), intramedullary intervertebral disc extrusion (IIVDE) and, more recently, far-lateral disc extrusion (8–12). Cervical IVDD in cats is less frequently reported compared to thoracolumbar and lumbar IVDD (13, 14), with only a limited number of studies specifically addressing cervical involvement, including isolated case reports and small case series describing ANNPE, IVDE and IVDP, managed both conservatively and surgically (9, 10, 13).

This case report describes the clinical presentation, MRI findings, and the 12-month outcome of a British Shorthair cat with multiple compressing cervical IVDDs and treated with double adjacent ventral slots, with the aim of adding more data on this disease in feline patients.

## Case description

**Figure 1:**
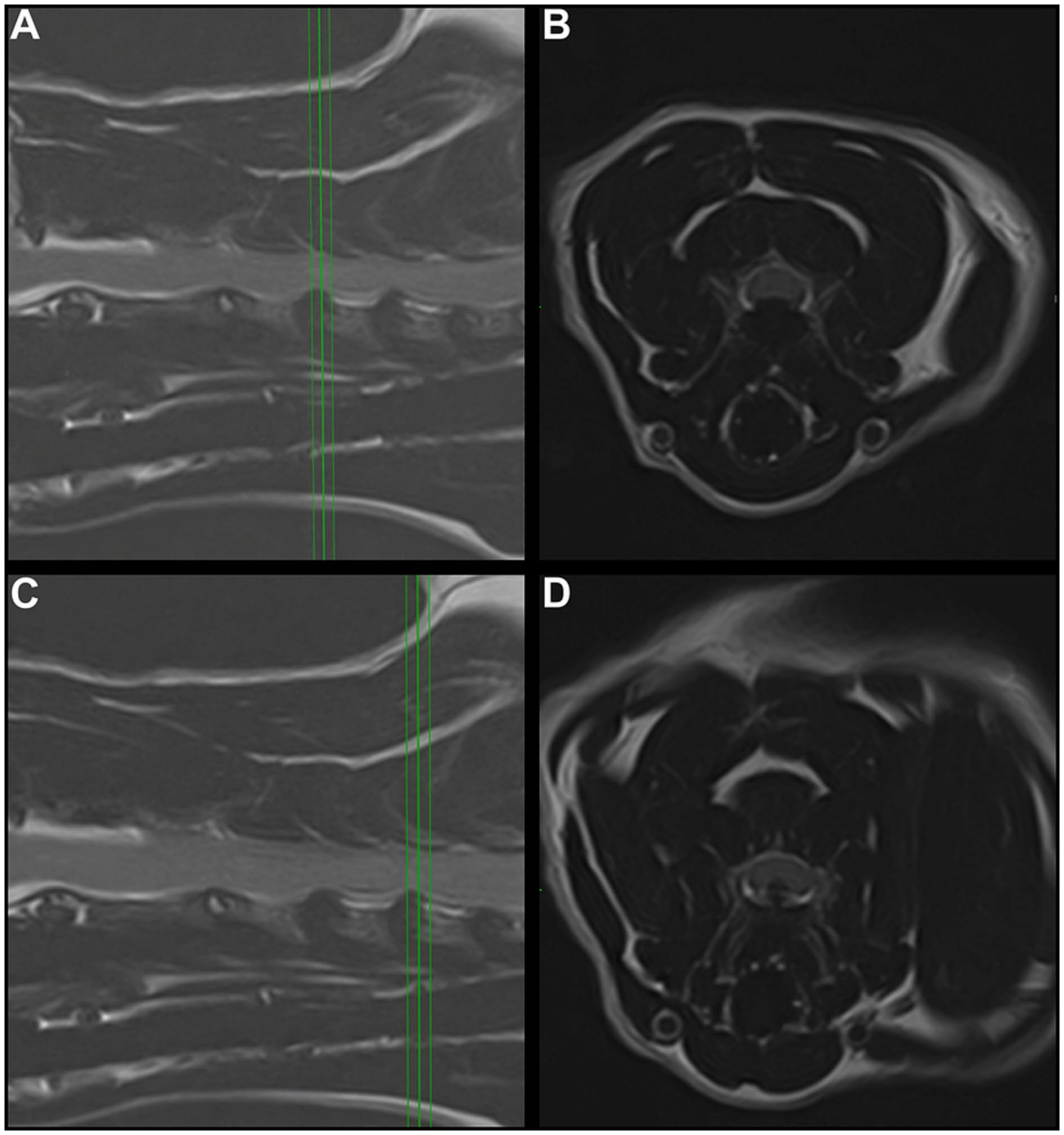
T2-weighted slightly parasagittal **(A,C)** and transverse **(B,D)** MRI of the cervical spine. Moderate intervertebral disc-associated extradural compressive lesions were identified at the levels of C3-C4 **(A,B)** and C4-C5 **(C,D)**. At both levels, a broad-based dorsal annular protrusion of disc material is present, resulting in ventral extradural compression of the spinal cord. The compressive material appears continuous with the parent intervertebral disc, with no evidence of free disc fragments or cranial or caudal migration, supporting a diagnosis of disc protrusion rather than extrusion. The spinal cord at these levels is dorsally displaced and mildly flattened.

**Figure 2:**
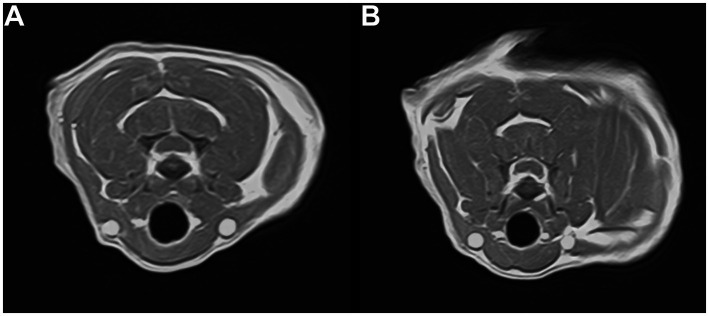
Transverse T1-weighted post-contrast MRI at the level of C3-C4 **(A)** and C4-C5 **(B)**. Homogeneous contrast enhancement of the extradural compressive material is observed.

An 8-year, 10-month-old spayed female British Shorthair cat was referred to the Neurology and Neurosurgery department at Paragon Referrals for investigation and treatment of chronic and progressive proprioceptive ataxia of all four limbs. The cat had a history of slowly progressive gait abnormalities beginning with pelvic limb ataxia approximately 4 months before referral. No investigations had been performed prior to presentation. Conservative management, including restricted activity and analgesia (meloxicam, Rheumocam 0.05 mg/kg SID, Chanelle Pharma), had been attempted for 2 months prior referral, with an initial marginal improvement and consequent further deterioration. At presentation, the cat was overweight (4.42 kg), with a body condition score of 7/9 (15). Clinical examination was unremarkable. Neurological examination showed non-ambulatory left-sided hemiparesis. Hopping responses on the left limbs were consistently delayed, lacking the smooth quality observed in the right limbs, when the cat was adequately supported. Paw placement was moderately reduced in the left thoracic and severely reduced in the left pelvic limbs. Spinal reflexes and withdrawal responses were unremarkable. Cranial nerves examination was unremarkable and no spinal hyperaesthesia was elicited. Based on the asymmetrical deficits affecting all four limbs and the absence of cranial nerve abnormalities, neuroanatomical localisation was consistent with a lesion affecting the cervical spinal cord between the C1 and C5 spinal cord segments. Blood tests (haematology and biochemistry) showed a decreased platelet count of 45·10^9^/L [reference interval (RI) 160–600], with a manual count of 1-2/hpf (although marked clumping was present), and mild increase in ALT 112 U/L (RI 0–100), BUN 14.13 mmoL/L (RI 5.40–11.40) and TP 81 g/L (60–80). A buccal mucosa bleeding time (BMBT) test was performed due to the low platelet count, which was normal (2.15 min; reference interval 1.5 to 3 min). The complete blood count was not repeated, as marked platelet clumping was present on the blood smear and the normal BMBT reduced concern for clinically relevant thrombocytopenia. The patient was premedicated with butorphanol 0.25 mg/kg (Torbugesic 10 mg/mL, Zoetis) and dexmedetomidine 2.5 μg/kg (Dexodomitor 0.5 mg/mL, Zoetis) intravenously (IV), induced with alfaxalone 0.3 mg/kg IV (Alfaxan 10 mg/mL, Jurox) and general anaesthesia (GA) was maintained with isoflurane (IsoFlo, Zoetis) in oxygen. Magnetic resonance imaging (MRI) of the cervical spine was performed with a 1.5 T scanner (Toshiba). Sagittal and transverse T2- and T1-weighted sequences were acquired, along with post gadolinium sagittal, transverse T1-MPRAGE and dorsal 3DT1-weighted sequences. MRI revealed multiple disc-associated extradural compressive lesions, producing moderate ventral compression of the spinal cord at the C3-C4 and C4-C5 levels, and mild ventral compression at the C5-C6 level. At the dorsal aspects of the affected discs, hypointense material accumulation extended within the ventral aspect of the vertebral canal, attenuating the ventral part of the epidural fat and subarachnoid space, causing deformation of the spinal cord. The adjacent spinal cord appeared of normal signal intensity while the dorsal subarachnoid space and the epidural fat remained visible. On post-gadolinium images, peripheral enhancement of the compressive extradural material was present at the C3-C4 and C4-C5 levels. Cerebrospinal fluid collection was not performed due to the presence of compressive cervical spinal lesions, the associated risk of clinical deterioration, and the limited anticipated diagnostic benefit in this case, given the structural abnormalities identified on MRI (see Figures 1, 2).

**Figure 3:**
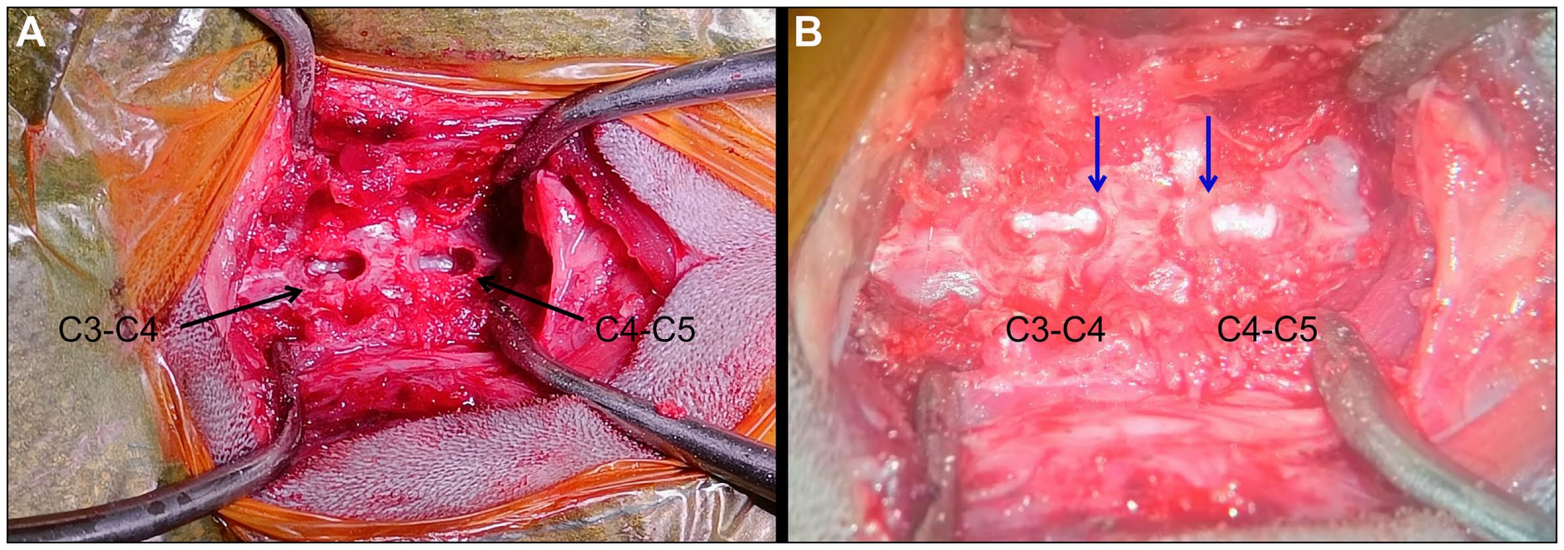
Intraoperative picture of both completed ventral slots at C3-C4 and C4-C5 levels without **(A)** and with intraoperative microscope **(B)**. The rectangular windows look symmetrical, with slightly reduced length of both slots on the C4 vertebra (blue arrows). The spinal cord appears in a normal position with no residual compression.

Due to the progression of clinical signs, surgical decompression was advised. The following day, the patient was premedicated with medetomidine 5 μg/kg (Sedator 1 mg/mL, Zoetis) and methadone 0.2 mg/kg (Comfortan 10 mg/mL, Dechra) IV and GA was induced and maintained as previously described. The cat was surgically prepared and positioned in dorsal recumbency. The neck was extended using a sandbag underneath. A standard ventral midline mid-cervical approach was performed. Following blunt dissection and retraction of the ventral cervical musculature and major neck structures, the C3-C4 and C4-C5 spaces were identified and confirmed using intraoperative fluoroscopy. Ventral slot surgery was performed at both sites using a high-speed burr under magnification with a neurosurgical operating microscope (Zeiss OPMI CS NC-2, Germany). Each slot was centred on the vertebral midline and extended dorsally to the level of the dorsal annulus fibrosus. Care was taken to create the rectangular slot windows that did not exceed 25%–30% of the width of the vertebral bodies, allowing appropriate access the spinal canal (16). Due to concerns regarding the structural implication of a double ventral slot on the C4 vertebra, even when adhering to the one-third length rule, the length of both slots was reduced, caudally at C3-C4 and cranially at C4-C5, to prevent over-weakening of C4, instability and/or iatrogenic fracture. At both levels, solidified nucleus pulposus material with dorsally thickened annulus fibrosus was removed. The slot sites were fully explored on the lateral, cranial and causal sides using a blunt curved nerve hook to ensure complete spinal cord decompression, with the cord sitting in normal position (Figure 3). Lavage was subsequently used to remove any debris, and the surgical site was routinely closed with 3-0 Polydioxanone (PDS II, Ethicon) for the muscle and fascia, and 4–0 Poliglecaprone 25 (Monocryl, Ethicon) for the subcutaneous and intradermal layers. No samples were collected for histopathological analysis.

The patient recovered uneventfully, and an indwelling five French Foley (MILA international) urinary catheter was placed to aid recovery. The cat was initially non-ambulatory tetraparetic, but gradually improved and was discharged 3 days post-operatively with an ongoing course of gabapentin 25 mg (BOVA) orally, every 8 hours. The cat was fully ambulatory 10 days after surgery. At the one-month follow-up, the neurological examination showed ambulatory paraparesis, lateralising to the left, with moderately reduced paw placement and severely reduced extensor thrust on the left pelvic limb. Physiotherapy was advised at this stage. At the two-month follow-up, the neurological status had improved, and the patient showed only left-sided pelvic limb monoparesis with mildly reduced postural reaction on the left pelvic limb. A final follow-up was performed 12 months postoperatively, which revealed a mild left pelvic limb monoparesis and minimal reduction in postural reaction on the left pelvic limb. The patient is currently undergoing long-term physiotherapy at home.

## Discussion

Cervical IVDD is still rarely reported in cats, and it accounts for approximately 5% of all feline IVDD cases (9, 10, 13, 14). The available literature is limited, and, due to paucity of reports, there is still scarce evidence regarding the optimal management of cervical feline IVDD. Historically, radiography was used to investigate spinal changes in cats, with an estimated prevalence of 0.46 to 4.4% (17). With the widespread availability of advanced imaging modalities, such as MRI, which is much more sensitive to spinal parenchyma and disc changes (9, 18), the prevalence has risen. The latest data shows that 70.2% of cats with suspected myelopathy exhibit at least one degenerative spinal change (17). Therefore, disc degeneration is a frequent finding in the aging feline population, especially within the cervical segment (4, 5, 18). This age-related degradation of the structural integrity of the disc predisposes these patients to IVDP, which may present with a broad spectrum of clinical signs from subclinical to chronic progressive non-ambulatory tetraparesis (2, 3, 8). Most of these lesions were located in the thoracolumbar region, with cervical lesions being notably less common; only one case of cervical IVDP (C3-C4) was identified, while several other cervical cases were classified as ANNPE (8). A 2024 post-mortem study using the modified Pfirrmann grading system to investigate the prevalence of IVDD, found that among 1,544 discs in asymptomatic cats, 42.7% showed signs of degeneration (Grade 2), though only 15.2% reached higher severity (Grade 3) (5). Specifically, within the cervical spine, MRI evidence shows that disc degeneration is commonly identified in asymptomatic cats; however severe or advanced degeneration is relatively uncommon (5). Although this study did not directly assess neurological deficits, it suggests that most age-related cervical disc changes are mild to moderate. This is further supported by evidence suggesting that while IVDE is clinically significant in 100% of patients, IVDP results in neurological signs in only 9% of cats where it is identified (17). Our patient belongs to that rare 9% pool, illustrating that cervical IVDP, can occasionally produce severe, progressive, non-ambulatory hemiparesis and tetraparesis. The MRI findings of the described case were characterised by a broad-based, smooth ventral bulge of disc material in direct continuity with the parent disc, consistent with an intact annulus fibrosus, a marked reduction in T2-weighted signal intensity and moderate-to-severe ventral spinal cord compression (19, 20), which directly corresponded to the patient’s neurological deficits. The presence of multiple instead of single disc herniations, chronic morphological features and the lack of extradural dispersed material were more likely to be associated with protrusions rather than extrusions. While, as previously mentioned, IVDE is statistically more likely to cause clinical signs (17), the focal nature of the compression in this patient suggested that the degenerative protrusions were the primary driver of the neurological dysfunction. This case serves as a reminder that while most feline cervical protrusions are incidental, cervical IVDD cannot be dismissed when the neuroanatomical localisation suggests otherwise. Both conservative and surgical management have been described to treat IVDD in cats, depending on lesion type, severity of neurological signs and disease progression (1, 7, 10). Evidence supporting conservative management of feline IVDD is extremely limited, with most published outcome data deriving from surgically treated cats; conservative management was reported infrequently and primarily in thoracolumbar disease (1, 7). Reports describing conservative management of cervical IVDD in cats are restricted to an isolated case report (9). Consequently, conclusions regarding the efficacy of conservative management for feline cervical IVDD remain difficult to draw. Management strategies may also differ between IVDE and IVDP, or more broadly between acute and chronic disc-associated compressions; however, no strict evidence-based recommendations currently exist in feline patients. Clinical decision making is usually driven by neurological severity, progression, and degree of compression rather than the lesion subtype alone. The ventral slot procedure is the most described surgical technique for ventral cervical spinal cord decompression in small animals (21), and it is indicated when persistent pain (which might be challenging to assess in cats) and muscle fasciculation are present, medical management failed, or when the patient presents with severe neurological deficits (22). While it is largely described in dogs, only four cases are reported in cats, one of which resulted in postoperative death due to respiratory arrest (10, 13). Nevertheless, a recent review suggests the prognosis for feline IVDD is generally favourable, regardless of the treatment choice, with an approximately 85% of cases achieving a functional recovery (14). In cases of multilevel cervical compression, multiple site ventral slots decompressions have been described, with favourable outcomes, in canine patients and one feline patient (10, 23–26). From a structural point of view, when performing ventral slots, postoperative vertebral instability or subluxation is the primary risk (27). Studies investigating ventral slot dimensions and safety margins, with recommendation of being not bigger than the 33% of the vertebral width (28), as well as those proposing modified cranially shifted slot techniques to reduce postoperative instability (23), have been conducted in canine patients. At present, comparable data are lacking in cats, which is clinically relevant when extrapolating surgical guidelines across species with differing cervical spine anatomy and biomechanical characteristics. In addition to these structural considerations, haemorrhage represents a recognised intraoperative complication due to proximity of regional vascular structures, although it can be mitigated with appropriate positioning and meticulous surgical technique (21). Given all these potential risks, careful preoperative planning is essential, as it directly influences surgical strategy in cases of multiple compressing IVDD. While an alternative surgical strategy in dogs with multilevel disease involves addressing only the single most clinically relevant acute disc, with favourable outcomes (29), it remains unknown whether such approach is applicable to cats with multilevel cervical IVDP. In this case, we argue that a single-site decompression would have left significant residual compression at the adjacent level, potentially hindering recovery or even worsening the remaining lesion due to altered postoperative biomechanics. By addressing both affected discs, we ensured a complete decompression of the spinal cord and mitigated the risk of the non-addressed protrusion becoming a source of persistent pain or progressive neurological decline. Three out of five cats reported in veterinary literature, treated with a ventral slot procedure (including the presented case), are British Shorthair cats. While in previous studies the prevalence of IVDD is shown to be higher in Domestic Shorthair cats, this might be secondary to a population bias. In some breed of dogs, a genetic component is known to have a role in risk of IVDD (30–34) but in cats there is much less data. A 2017 study investigated the prevalence of thoracolumbar IVDD in cats in one institution and, between purebred cats, British Shorthair and Persians were overrepresented (6). Similarly, a study from 2020 found British Shorthair cats were overrepresented, between purebred cats, for thoracic vertebral canal stenosis (30). Genetic research of an inbreeding case in British Shorthair kittens showing severe spinal deformities, discovered a recessive gene, which mutation can cause a form of skeletal dysplasia (31). Further epidemiologic studies are warranted in this breed. Obesity is also a well-recognised and clinically significant risk factor for IVDD in humans (32) and in dogs (33). In contrast, current veterinary literature does not correlate increased body weight to IVDD in cats, although heavier body weight may be associated with increased spinal degenerative changes (17). This is likely due to fewer epidemiological studies in cats and discrepancy on individual BCS and body weight recording. Given the robust phenotype of the British Shorthair cat, these factors may have contributed to the clinically significant, multilevel cervical compression observed in this patient.

This report is limited by the absence of postoperative imaging, which precluded objective assessment of the degree of decompression and long-term spinal stability. In addition, no disc material was retrieved for histopathological analysis for definitive classification of the lesion subtype. Nevertheless, the favourable clinical outcome supports the clinical relevance of surgical decompression in cats with progressive multilevel cervical IVDD.

## Conclusion

Although cervical IVDD is uncommon in cats, the present case illustrated that surgical decompression using two adjacent ventral slots is feasible and can result in a favourable long-term outcome. While the use of ventral slot surgery is widely reported in dogs, its application in cats remains only sporadically reported. Performing a double ventral slot always raises a concern on vertebral instability and perioperative complications; however, in this case, two modified adjacent ventral slots were performed without complications or evidence of postoperative instability. These findings suggest that, with careful case selection and thorough preoperative planning, the technique may be safely performed in selected feline patients. Transient postoperative neurological deterioration may occur and should be anticipated, as it is consistent with previous reports. This article also raises a breed-related predisposition of British Shorthair cats to spinal diseases. While a population bias cannot be excluded, this breed has been overrepresented in several studies investigating various spinal diseases and are overrepresented in the cohort of case reports describing surgical management of cervical IVDD. Given the documented genetic mutation associated with skeletal dysplasia, potential hereditary factors influencing vertebral or disc morphology cannot be ruled out, and this warrants further investigations. Overall, surgical intervention of multiple cervical IVDP could be considered in cats presenting with progressive neurological deficits. However, given the limited available literature, further studies are required to better define indications, complication rates, and long-term outcomes in this species.

## Data Availability

The original contributions presented in the study are included in the article/supplementary material, further inquiries can be directed to the corresponding author.
